# A mathematical model to assess the effects of COVID-19 on the cardiocirculatory system

**DOI:** 10.1038/s41598-024-58849-3

**Published:** 2024-04-09

**Authors:** Andrea Tonini, Christian Vergara, Francesco Regazzoni, Luca Dede’, Roberto Scrofani, Chiara Cogliati, Alfio Quarteroni

**Affiliations:** 1 MOX, Dipartimento di Matematica, Politecnico di Milano, Milan, Italy; 2 LABS, Dipartimento di Chimica, Materiali e Ingegneria Chimica, Politecnico di Milano, Milan, Italy; 3 UOC Cardiochirurgia Fondazione IRCCS Ca’ Granda, Ospedale Maggiore Policlinico di Milano, Milan, Italy; 4 Internal Medicine, L. Sacco Hospital, Milan, Italy; 5 Department of Biomedical and Clinical Sciences, Università di Milano, Milan, Italy; 6 Institute of Mathematics, École Polytechnique Fédérale de Lausanne, Lausanne, Switzerland

**Keywords:** Biomedical engineering, Viral infection

## Abstract

Impaired cardiac function has been described as a frequent complication of COVID-19-related pneumonia. To investigate possible underlying mechanisms, we represented the cardiovascular system by means of a lumped-parameter 0D mathematical model. The model was calibrated using clinical data, recorded in 58 patients hospitalized for COVID-19-related pneumonia, to make it patient-specific and to compute model outputs of clinical interest related to the cardiocirculatory system. We assessed, for each patient with a successful calibration, the statistical reliability of model outputs estimating the uncertainty intervals. Then, we performed a statistical analysis to compare healthy ranges and mean values (over patients) of reliable model outputs to determine which were significantly altered in COVID-19-related pneumonia. Our results showed significant increases in right ventricular systolic pressure, diastolic and mean pulmonary arterial pressure, and capillary wedge pressure. Instead, physical quantities related to the systemic circulation were not significantly altered. Remarkably, statistical analyses made on raw clinical data, without the support of a mathematical model, were unable to detect the effects of COVID-19-related pneumonia in pulmonary circulation, thus suggesting that the use of a calibrated 0D mathematical model to describe the cardiocirculatory system is an effective tool to investigate the impairments of the cardiocirculatory system associated with COVID-19.

## Introduction

The coronavirus disease 2019 (COVID-19) caused by severe acute respiratory syndrome coronavirus 2 (SARSCoV-2) primarily affects the respiratory system, even if it does not spare other organs as it occurs for the cardiovascular system at large^1–3^. In severe COVID-19-related pneumonia, impairment of heart function seems to be mainly driven by right ventricle involvement, while consequences on the left ventricle appear to be less common^4^. Right ventricle dilation, diminished right ventricular function and elevated pulmonary arterial systolic pressure have been described and are associated with mortality in severe COVID-19^5,6^. Respiratory failure with shortening of oxygen supply represents the main clinical picture of the disease. Hypoxemia is associated with a huge increase in intrapulmonary shunt (measuring the percentage of blood that does not oxygenate in the lungs) due to alveolar fluid filling/consolidations. In fact, the pulmonary shunt fraction is in physiological conditions below 5%^7^, whereas it reaches values up to $60\mathrm{\%}$ in patients with ongoing COVID-19 infection^8,9^. Endothelial damage with diffuse micro-thrombosis has been widely described in histological studies in COVID-19 pneumonia patients and is associated with an increase in dead space in lungs and thus in non-oxygenated blood^10^. On the other hand, such an increase in intrapulmonary shunt has been postulated to depend also on an impairment of hypoxic pulmonary vasoconstriction that should restrict pulmonary flow to hypo-ventilated lung areas^11^. These mechanisms do not seem to be correlated with each other and seem to coexist to varying degrees in COVID-19 pneumonia patients^12^.

In this context, physics-based mathematical models are an effective and accurate tool for making predictions through virtual scenarios and for providing clinical answers in terms of impairments of the cardiovascular function associated with COVID-19^13–15^. In this respect, we previously studied, by means of a computational lumped-parameter (i.e. 0D) model, possible effects in terms of, e.g. cardiac output and pressures^16^. However, this previous study did not integrate clinical data into the analysis in a systematic manner.

The main novelty of this paper is to assimilate, by means of a calibration method, clinical data coming from measurements on COVID-19 patients regarding, e.g. cardiac volumes and vascular pressures, into the computational model proposed in ref.^17^ to make it patient-specific and then to use such calibrated model for making predictions on the impairments of the cardiovascular function associated with the ongoing infection. To do this, we improved the model of ref.^17^ by adding further compartments representing systemic and pulmonary micro-vasculatures. In this work, we focused on reproducing the blunted hypoxic pulmonary vasoconstriction, that is among the causes of the reduction in blood oxygenation, by means of the calibration of the model, thus neglecting the possible increase in pulmonary resistance associated to diffuse micro-thrombosis.

Our final goal is to study possible associations between the ongoing infection of COVID-19 and the impairments on the cardiocirculatory system by estimating physical quantities of clinical interest not available as measured clinical data, e.g. pulmonary arterial and capillary wedge pressures, heart chamber blood volumes and pressures, and by performing a statistical analysis on these quantities.

## Methods

The modified lumped-parameter model consists of a system of ordinary differential equations (ODEs) that needs to be numerically solved to allow the computation of different model outputs of clinical interest. We calibrated the model to fit some clinical data of patients hospitalized for severe COVID-19-related pneumonia in the Internal Medicine ward of L. Sacco Hospital in Milan, Italy, between March and April 2020. We analysed the statistical reliability of the model outputs for each successful calibration by means of uncertainty intervals and, finally, we performed a statistical analysis on clinical data or model outputs by means of hypothesis tests to highlight the impairments of the cardiocirculatory system associated with COVID-19 pneumonia.

We identified four groups of quantities, taken from the dataset or obtained as an output of the calibrated model:

(i) The *clinical data* used for the model calibration, obtained from clinical measurements and referring to physical quantities (PQ1), as, for example, the maximal left atrial volume (LA_Vmax_) and the systolic systemic pressure (SAP_max_);
(ii) The *inputs* of the model (heart rate HR and body surface area BSA) and of the calibration procedure (right ventricular fractional area change RV_FAC_ and tricuspid annular plane systolic excursion TAPSE), provided by other clinical measurements;
(iii) The parameters of the model (e.g. resistances and compliances) determined through a calibration procedure, from now on referred to as *calibrated parameters*;
(iv) The outputs of the numerical simulation of the model (e.g. flow rate and mean pressure), from now on referred to as *model outputs*. Some of them (MO1) referred to physical quantities (PQ1) that were also measured (clinical data), for example, LA_Vmax_ and SAP_max_. Other model outputs (MO2) referred to physical quantities (PQ2) that were not measured but quantified only by means of the computational model. Examples of the latter are the mean left atrial pressure (LA_Pmean_) and indexed right ventricular end diastolic volume (RV_I-EDV_). The complete list of PQ1 and PQ2 is reported in Supplementary Table 1.

We remark that the indexed value of volumes of a patient can be computed dividing the volumes by the BSA of that patient (Supplementary Table 2). In what follows, an “I-” that precedes a subscript of a volume means that the volume is indexed (for example, LV_I-EDV_ is the indexed left ventricular end diastolic volume).

**Figure 1:**
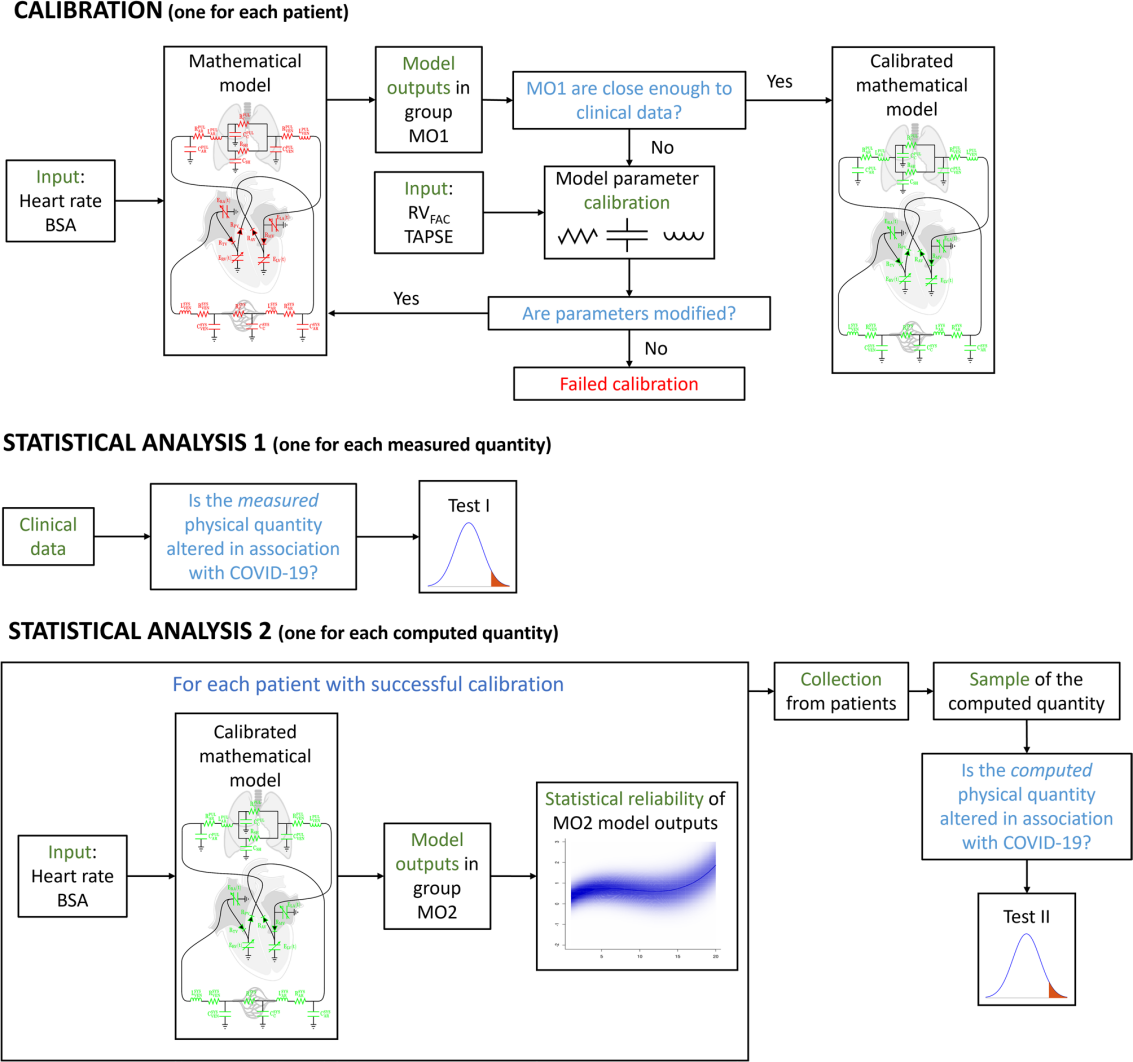
Diagram flowchart of the procedure used in this study. Top: calibration; mid: statistical analysis of measured physical quantities; bottom: statistical analysis of computed physical quantities. Calibration: the mathematical model required as inputs HR and BSA of a specific patient. The model computed MO1 using an initial setting of parameters (that could need to be calibrated, so they are highlighted in red). If MO1 were close enough to the clinical data the model was considered calibrated (the parameters are highlighted in green); if not, the calibration method was iteratively applied to the parameters using RV_FAC_ and TAPSE as inputs. If the parameters were not modified the calibration failed; if not, MO1 were recomputed by using the new setting of parameters and the previous steps were repeated. Statistical analysis 1: we performed hypothesis tests on clinical data (test I). Statistical analysis 2: HR and BSA were used as inputs of the calibrated model for every patient with a successful calibration, the model computed the MO2 and we checked the statistical reliability of MO2. We collected the reliable MO2 from every patient and we performed hypothesis tests on the reliable MO2 of all the patients (test II).

For the sake of clarity, we reported in Fig. 1 the diagram flowchart of the followed procedure that is described in detail in what follows.

### Dataset

The dataset consists of $58$ patients, who all required oxygen supplementation but none of them was on mechanical ventilation. Of such patients, only $29$ were calibrated according to point (iii) above (see Calibration subsection below) ($56\pm 18$ years). Such patients did not present symptoms or signs of heart failure or substantial structural cardiac disease; $10$ out of $29$ were older than $64$ years; $6$ patients had arterial hypertension, $1$ had diabetes and $4$ showed the association of hypertension and diabetes (Supplementary Table 2).

The echocardiography of each patient was performed early after the admission to the hospital. Examinations were performed at bedside using a Philips CX-50 portable device by expert operators. Measures were defined according to the latest European and American Echocardiography Society guidelines^18,19^.

Each patient provided consent to use his/her data for observational studies. The institutional board has approved the study with protocol number 16088/2020.

### Mathematical model

The cardiovascular system was studied by means of a lumped-parameter (0D) mathematical model that splits the system into compartments (e.g. right atrium, systemic arteries/veins) and, for each of them, the time evolution of model outputs (pressures, flow rates and cardiac volumes) is modelled by a system of ODEs^20,21^. The lumped-parameter model is described through an electrical circuit analogy: the current represents the blood flow through vessels and valves; the electric potential the blood pressure; the electric resistance plays the role of the resistance to blood flow; the capacitance represents the vessel compliance; the inductance the blood inertia; the increase in elastance the cardiac contractility.

**Figure 2:**
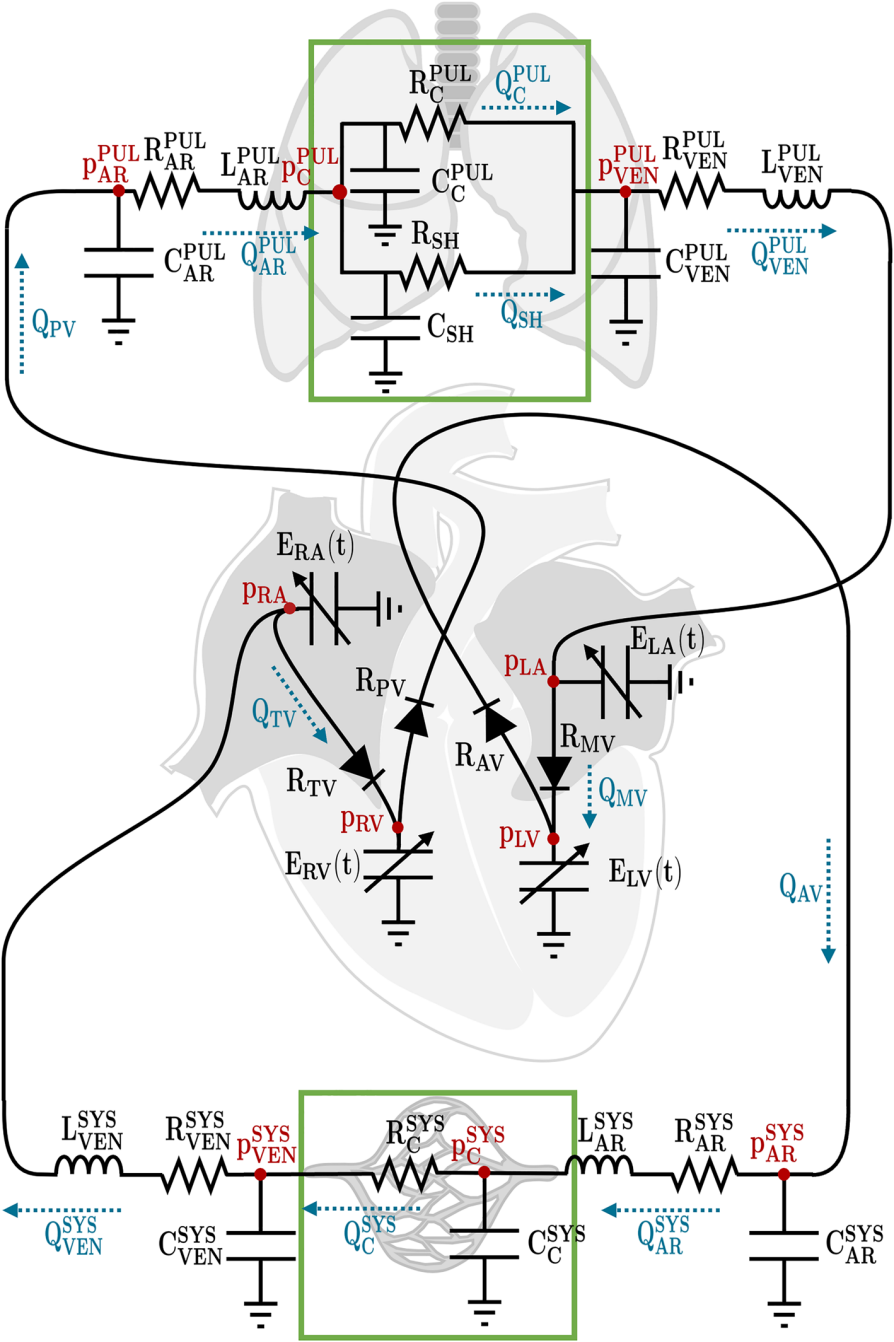
Lumped-parameter cardiocirculatory model. The unknown pressures and flow rates are in red and blue, respectively, whereas the model parameters are in black. Notice in the green boxes the new compartments with respect to^16^ featuring this work.

There are different possible choices and number of compartments, depending on the purpose of the study, for the construction of a lumped-parameter model (e.g. Refs.^16,17,22,23^). We considered the computational model introduced in Ref.^17^, wherein the four heart chambers, the systemic and pulmonary circulations, with their arterial and venous compartments were included, and we substituted the 3D left ventricle with a 0D component (as in Ref.^16^) and we added two new compartments accounting for systemic and pulmonary capillaries. The pulmonary capillary circulation was also split in two compartments accounting for oxygenated and non-oxygenated capillaries (Fig. 2).

The system of ODEs associated with the lumped-parameter model is formed by the equations representing continuity of flow rates at nodes and of pressures in the compartments, and its numerical solution allows to compute several model outputs as functions of time: the left and right atrial and ventricular volumes (${V}_{{\text{LA}}}$, ${V}_{{\text{LV}}}$, ${V}_{{\text{RA}}}$ and ${V}_{{\text{RV}}}$), the systemic and pulmonary arterial, capillary and venous pressures (${p}_{{\text{AR}}}^{{\text{SYS}}}$, ${p}_{{\text{C}}}^{{\text{SYS}}}$, ${p}_{{\text{VEN}}}^{{\text{SYS}}}$, ${p}_{{\text{AR}}}^{{\text{PUL}}}$, ${p}_{{\text{C}}}^{{\text{PUL}}}$ and ${p}_{{\text{VEN}}}^{{\text{PUL}}}$), the systemic and pulmonary arterial and venous blood fluxes (${Q}_{{\text{AR}}}^{{\text{SYS}}}$, ${Q}_{{\text{VEN}}}^{{\text{SYS}}}$, ${Q}_{{\text{AR}}}^{{\text{PUL}}}$ and ${Q}_{{\text{VEN}}}^{{\text{PUL}}}$).

Starting from these functions, it is possible to compute the pressures of the four cardiac chambers (${p}_{{\text{LA}}}$, ${p}_{{\text{LV}}}$, ${p}_{{\text{RA}}}$ and ${p}_{{\text{RV}}}$), the blood fluxes through the valves (${Q}_{{\text{MV}}}$, ${Q}_{{\text{AV}}}$, ${Q}_{{\text{TV}}}$ and ${Q}_{{\text{PV}}}$), through the systemic capillaries (${Q}_{{\text{C}}}^{{\text{SYS}}}$) and through oxygenated and non-oxygenated pulmonary capillaries (${Q}_{{\text{C}}}^{{\text{PUL}}}$ and ${Q}_{{\text{SH}}}$), and all the model outputs referring to PQ1 and PQ2 (Supplementary Table S1).

We considered reference values of the parameters (such as resistances and compliances) such that all the model outputs were in the reference healthy ranges of the corresponding physical quantities taken from the literature^7,18,19,24^ for an ideal individual with HR equal to $80$ bpm (beats per minute) and BSA equal to $1.79$ m^2^ (Supplementary Table S3). We did not consider model outputs computed starting from the flow rates, because they are not uniquely defined depending on the tract of the compartment where they are measured, from ${p}_{{\text{C}}}^{{\text{SYS}}}$, due to the heterogeneity of the pressures of systemic capillaries among tissues, and from ${p}_{{\text{VEN}}}^{{\text{SYS}}}$, even if we recovered the value of central venous pressure, that coincides with the right atrial pressure^24^.

We reported the system of ODEs associated with the lumped-parameter model in Supplementary Equations S1. The lumped-parameter model was numerically discretized by means of Dormand-Prince method^25^ (adaptive stepsize Runge–Kutta) which was implemented in Python using the Jax library^26^.

### Calibration

The lumped-parameter model was characterized by parameters representing the functional properties of the compartments (e.g. resistances). To properly select such values for a specific compartment and patient, a calibration procedure was needed^27,28^.

We chose a priori the cardiac timings and the resistance of oxygenated pulmonary capillaries (${{\text{R}}}_{{\text{C}}}^{{\text{PUL}}}$) equal to the associated reference values. In particular, we fixed ${{\text{R}}}_{{\text{C}}}^{{\text{PUL}}}$ to avoid modelling micro-thrombosis because of its possible increase. For the remaining parameters, the calibration of the model relied on the method we presented in Ref.^27^, that is aimed to reduce the sum of squared relative errors between the model outputs MO1 and clinical data, modifying the parameters of the model in suitable bounded intervals ${{\text{I}}}_{{\text{i}}}$, for $i=1,\dots ,{N}_{{\text{p}}}$, where ${N}_{{\text{p}}}$ is the number of parameters, independent of the patient, built starting from the reference values of parameters mentioned before (Supplementary Table S3). Specifically, we chose to calibrate those parameters among the latter according to a sensitivity analysis estimating the absolute correlation coefficients between parameters and model outputs (Supplementary Table S4). We calibrated only the parameters featuring at least one absolute correlation coefficient greater than $0.1$ that was associated to provided clinical data. To reproduce the blunted hypoxic pulmonary vasoconstriction condition, the resistance of non-oxygenated pulmonary capillaries (${{\text{R}}}_{{\text{SH}}}$) could decrease in such a way that the shunt fraction could reach values up to $70\%$ in the worst-case scenario. The list of amendable parameters varies between different patients according to the different clinical data provided.

The calibration was based on clinical measurements of COVID-19 patients that were provided by L. Sacco Hospital in Milan and referred to HR and BSA, which were used as inputs for the lumped-parameter model, RV_FAC_ and TAPSE, which determined the bounded interval ${{\text{I}}}_{\overline{{\text{i}}} }$ used during the calibration, with $\overline{i }$ the index referring to the right ventricular active elastance, and the clinical data, given by a subset of the pressures and volumes involved in the cardiac circulation (Supplementary Table S2).

To provide further mathematical details, we indicate with $\mathbf{p}$ a configuration of parameters of the cardiocirculatory model. The calibration method aimed to find the configuration of parameters ${\overline{\mathbf{p}} }^{{\text{j}}}$ which minimized the loss function for the specific patient $j$, that reads:

1 $$L^{{\text{j}}} \left( {\mathbf{p}} \right) = \sum\limits_{{{\text{l}} = 1}}^{{{\text{N}}^{{\text{j}}} }} {\left( {\frac{{q_{{{\text{m}}_{{\text{j}}} \left( {\text{l}} \right)}}^{{\text{j}}} \left( {\mathbf{p}} \right) - d_{{\text{l}}}^{{\text{j}}} }}{{d_{{\text{l}}}^{{\text{j}}} }}} \right)^{2} } ,$$

where ${N}^{{\text{j}}}$ is the number of available echographic clinical data for patient $j$, ${d}_{{\text{l}}}^{{\text{j}}}$ is the value of the l-th clinical data of patient $j$ (Supplementary Table S2) and ${q}_{{{\text{m}}}_{{\text{j}}}\left({\text{l}}\right)}^{{\text{j}}}$ is the value of the model output related to the l-th clinical data of patient $j$. The index $m$ of ${q}_{{\text{m}}}^{{\text{j}}}$ lies in $\{1,\dots ,{N}_{{\text{q}}}\}$ where ${N}_{{\text{q}}}$ is the number of both MO1 and MO2. We considered the model calibrated for a specific patient if the loss function was below ${10}^{-3}$. Notice that, for some patients, the calibration procedure could fail, if, for example, it reaches the minimum of the loss function that is above the required threshold.

Moreover, to improve the robustness of the calibration procedure, we repeated, for every patient, the calibration three times, with different initial configurations of parameters, and we considered the calibrated setting of parameters that returned the lowest loss function. As anticipated above, only $29$ out of $58$ patients were successfully calibrated. We noticed that by performing $4$ times the calibration procedure the number of calibrated patients was still equal to $29$, precisely as after $3$ calibrations.

The loss function (1) was minimized by the Quasi-Newton method L-BFGS-B^29^ implemented in Scipy by computing its gradient by means of automatic differentiation (reverse mode gradient) included in the library Jax^26^.

### Uncertainty intervals

For every patient $j$ calibrated with a loss function below ${10}^{-3}$, a configuration of parameters ${\overline{\mathbf{p}} }^{{\text{j}}}$ was at disposal. The loss function was computed using the clinical data provided by L. Sacco Hospital, which were related to *measurement errors* (Supplementary Table S1), that also affected the uncertainty of the model outputs ${\mathbf{q}}^{{\text{j}}}$. We needed to determine, for every patient, if the related model outputs were reliable or not, so we proceeded along two steps:

1. Build a sample of candidate model outputs ${\mathbf{q}}^{{\text{j}},{\text{k}}}$ for $k=1,...,n$ ($n$ was $100$);
2. Determine, by employing a simple statistical analysis, whether the mean of the model outputs was reliable.

Regarding step 1, for every provided clinical data ${d}_{{\text{l}}}^{{\text{j}}}$ of patient $j$, we built an interval ${{\text{M}}}_{{\text{l}}}^{{\text{j}}}$ centred in the value of the clinical data with width equal to two times the measurement error (Supplementary Table S1). Then, we built the samples ${\mathbf{q}}^{{\text{j}},{\text{k}}}$ by following the subsequent procedure:

- Choose a relative width $w$ ($w$ was $12.5\%$);
- Build an interval centred at ${\overline{p} }_{{\text{i}}}^{{\text{j}}}$ and with width $2w{\overline{p} }_{{\text{i}}}^{{\text{j}}}$ for every $i=1,\dots ,{N}_{{\text{p}}}$. If this interval is not included in the parameter interval ${{\text{I}}}_{{\text{i}}}$ used for the calibration, then cut off its overflowing extremities.
- Perturb every parameter of the calibrated patient sampling from a uniform distribution in the corresponding interval built at point b) thus obtaining ${p}_{{\text{i}}}^{{\text{j}}}$;
- Run a simulation of the cardiocirculatory model with parameters ${\mathbf{p}}^{{\text{j}}}$;
- Check if the model output ${q}_{{{\text{m}}}_{{\text{j}}}\left({\text{l}}\right)}^{{\text{j}}}$ generated at point d) lie in the intervals ${{\text{M}}}_{{\text{l}}}^{{\text{j}}}$. If they do, save the new configuration of acceptable model outputs ${\mathbf{q}}^{{\text{j}}}$, otherwise reject it;
- Repeat from point c) until $n$ iterations are performed;
- Check if the acceptance ratio (ratio between the number of saved configurations and the number of iterations) is within $[0.1, 0.15]$. If it does, repeat from point c) to e) until $n$ configurations are accepted because at this step the sample size of candidate model outputs is small (with $n=100$, the size is between $10$ and $15$), otherwise increase or decrease $w$ to retrieve the condition on the acceptance ratio, discard the previous configurations and repeat from point b).

Once the above procedure was concluded, we proceeded with step 2 by using the $n$ samples of acceptable model outputs ${\mathbf{q}}^{{\text{j}},{\text{k}}}$ for $k = 1, \dots , n$ generated at the previous step, for every specific patient $j$. If the standard deviation of the sample of a model output of patient $j$ was lower than 5% of its mean, we considered the mean reliable and we used it for the hypothesis tests. In this way, for every model output we built a sample of accepted values (depending on the patient), where sample size depended on the considered model output.

Prediction intervals could have been used for this analysis, but, if the sample was not normally distributed, a link function would be needed to retrieve normality^30^. We checked, for every patient $j$ and for every model output, if the sample of that model output was normally distributed by means of a chi-squared test. It turned out that the sample is not normally distributed for all patients. Thus, since we wanted to use the same statistical approach for every patient, we resorted to this heuristic approach based on standard deviation instead of prediction intervals.

### Statistical analysis

If the sample mean, calculated over all patients, of a clinical data or MO2 (referring to physical quantities PQ1 and PQ2, respectively) fell inside the healthy range of the corresponding physical quantity^7,18,19,24^, we did not consider the physical quantity altered in association with COVID-19 infection, otherwise we performed hypothesis tests to check whether the mean was significantly (p-value below $0.01$) increased or decreased with respect to the healthy range to investigate the impairments of the cardiovascular system in association with COVID-19 infection. If the sample mean, calculated over all patients, was less than the lower bound of the healthy range, the null hypothesis was that the mean was greater or equal than the lower bound of the healthy range, whereas the alternative hypothesis was that the mean was smaller than the lower bound of the healthy range. If we accepted the null hypothesis, then the corresponding physical quantity was considered not altered in association with the infection of COVID-19; otherwise, we considered the physical quantity altered in association with COVID-19. If, instead, the sample mean was greater than the upper bound of the healthy range, we proceeded similarly.

For each clinical datum, we computed the mean and the standard deviation of its sample without resorting to the mathematical model. The sample sizes were large enough to use one-tailed z-tests (assuming the variance equal to the unbiased sample variance) comparing their means to the nearest bound of the healthy range (test I).

For every MO2 we computed the mean and the standard deviation of its sample. We performed a chi-squared test and not every sample was normally distributed, so we opted for one-tailed z-tests (assuming the variance equal to the unbiased sample variance) only if the sample had more than $24$ elements comparing their means to the nearest bound of the healthy range (test II).

Notice that for group PQ1 the statistical analysis was carried out directly using the clinical data and not the MO1 values. Accordingly, the clinical data were used in a twofold way:

(i) To statistically compare PQ1 clinical measures with healthy ranges independently of the application of the proposed lumped-parameter model (test I);
(ii) To calibrate the lumped-parameter model for the patients at hand thus allowing to obtain MO2 that are statistically compared with healthy ranges (test II).

## Results

### Time transients of model outputs

**Figure 3:**
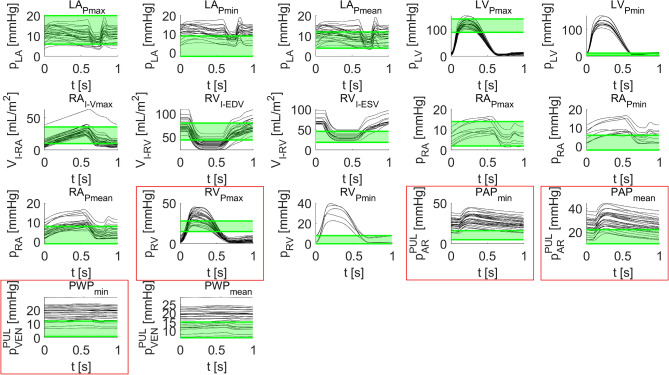
Time transients of model outputs during a cardiac cycle. In green, the reference healthy ranges of the corresponding PQ2 (the name reported above each graph) are highlighted. In red boxes the model outputs that possibly lie significantly outside of the healthy range are reported. The duration of a heartbeat was normalized to $1$ s. Notice the different sample size of the plots depending on the corresponding discarded patients. The model outputs plotted are the left atrial and ventricular pressures (${{\text{p}}}_{{\text{LA}}}$ and ${{\text{p}}}_{{\text{LV}}}$), the indexed right atrial and ventricular volumes (${{\text{V}}}_{{\text{I}}-{\text{RA}}}$ and ${{\text{V}}}_{{\text{I}}-{\text{RV}}}$), the right atrial and ventricular pressures (${{\text{p}}}_{{\text{RA}}}$ and ${{\text{p}}}_{{\text{RV}}}$), the pulmonary arterial and venous pressures (${{\text{p}}}_{{\text{AR}}}^{{\text{PUL}}}$ and ${{\text{p}}}_{{\text{VEN}}}^{{\text{PUL}}}$).

To perform a qualitative analysis, in Fig. 3 we reported the time-dependent model outputs (by normalizing the heartbeat duration) together with the healthy ranges (in green) related to the following physical quantities among PQ2: maximal, minimal and mean left atrial pressures (LA_Pmax_, LA_Pmin_ and LA_Pmean_), maximal and minimal left ventricular pressures (LV_Pmax_ and LV_Pmin_), indexed maximal right atrial volume (RA_I-Vmax_), indexed right ventricular end diastolic and systolic volumes (RV_I-EDV_ and RV_I-ESV_), maximal, minimal and mean right atrial pressures (RA_Pmax_, RA_Pmin_ and RA_Pmean_), maximal and minimal right ventricular pressures (RV_Pmax_ and RV_Pmin_), minimal and mean pulmonary arterial pressures (PAP_min_ and PAP_mean_) and the minimal and mean pulmonary wedge capillary pressures (PWP_min_ and PWP_mean_). Notice that, for each graph, only patients such that the corresponding model output had been found to be statistically reliable (on the basis of the estimated uncertainty interval) were reported. We point out that, from Fig. 3, some sample sizes were too small to analyse the corresponding model output (e.g., RV_Pmin_). For the remaining model outputs, we moved on to the statistical analysis to study the impairments of the cardiocirculatory system associated with COVID-19, as detailed in the next paragraph.

### Statistical analysis of PQ1 (clinical data, test I) and PQ2 (test II)

We analysed the PQ1 using the clinical data (test I—Table 1), namely: the maximal left atrial volume (LA_Vmax_), the left ventricular end diastolic and end systolic volumes (LV_EDV_ and LV_ESV_), the left ventricular ejection fraction (LV_EF_), the maximal right atrioventricular pressure gradient (max ∇p_rAV_), the systolic and diastolic systemic pressures (SAP_max_ and SAP_min_) and the systolic pulmonary pressure (PAP_max_). There was no statistical evidence that the clinical data related to PQ1 were altered in association with COVID-19-related pneumonia because the mean of the samples lied in the corresponding healthy ranges.

**Table 1:** Statistics of clinical data.

PQ1	Healthy range	Mean ± std dev
LA_I-Vmax_ [mL/m^2^] (LA_Vmax_ [mL])	[16, 34]^18^	32.7 ± 13.7 (n = 56), (59.1 ± 26.2)
LV_I-EDV_ [mL/m^2^] (LV_EDV_ [mL])	[50, 90]^24^	56.5 ± 11.6 (n = 58), (101.5 ± 24.1)
LV_ESV_ [mL]	[18, 52]^18^	36.8 ± 15.3 (n = 57)
LV_EF_ [%]	[53, 73]^18^	64.5 ± 7.5 (n = 58)
max ∇p_rAV_ [mmHg]	–	23.0 ± 5.9 (n = 42)
SAP_max_ [mmHg]	[–, 140]^18^	120.6 ± 14.7 (n = 58)
SAP_min_ [mmHg]	[–, 80]^18^	71.0 ± 11.4 (n = 58)
PAP_max_ [mmHg]	[15, 28]^24^	27.9 ± 5.1 (n = 40)

The mean and standard deviation of the samples are provided together with the sizes in brackets. Notice that the sample sizes of the clinical data are different due to heterogeneous samples. The hypothesis tests were not performed because the mean of the samples lied in the respective healthy range, Test I.

Instead, we analysed the PQ2 using the model outputs MO2 (test II—Table 2), obtaining the following outcomes:

I. For RV_Pmax_, PAP_min_, PAP_mean_, PWP_min_ and PWP_mean_ we rejected the null hypothesis and thus these physical quantities resulted significantly increased with respect to the healthy ranges;
II. For left ventricular stroke volume (LV_SV_), cardiac index (CI) and thus the cardiac output (CO), LA_Pmax_, LA_Pmean_, LV_Pmax_, RA_I-Vmax_, RV_I-EDV_, right ventricular ejection fraction (RV_EF_), systemic and pulmonary vascular resistances SVR and PVR, we did not reject the null hypothesis, thus there was no statistical evidence that these physical quantities were altered in association with COVID-19-related pneumonia;
III. The sample sizes of LA_Pmin_, LV_Pmin_, RV_I-ESV_, RA_Pmax_, RA_Pmin_, RA_Pmean_, RV_Pmin_ and the Shunt Fraction were too small to perform the hypothesis tests.

**Table 2:** Statistics of MO2.

	PQ2	Healthy range	Mean ± std dev	Test II (p-value)	COVID-19 literature ranges
Rejected null hypothesis	RV_Pmax_ [mmHg]	[15, 28]^24^	33.7 ± 6.8 (n = 29)	2.62E-06	[30, 46]^32^
	PAP_min_ [mmHg]	[5, 16]^24^	23.6 ± 6.2 (n = 29)	3.06E-11	[15, 26]^11^
	PAP_mean_ [mmHg]	[10, 22]^24^	27.1 ± 6.5 (n = 29)	9.97E-06	[25, 33]^11^
	PWP_min_ [mmHg]	[1, 12]^24^	17.1 ± 5.2 (n = 28)	1.04E-07	–
	PWP_mean_ [mmHg]	[6, 15]^24^	17.5 ± 5.1 (n = 28)	5.35E-03	[11, 18]^11^
Not rejected null hypothesis	LV_SV_ [mL]	[30, 80]^18^	74.0 ± 10.7 (n = 29)	–	[68,105]^11^
	CI [L/min/m^2^] (CO [L/min])	[2.8, 4.2]^24^	3.2 ± 0.5 (n = 29) (5.9 ± 1.0)	–	[2.7, 4.5]^11^/[1.98, 3.32]^33^ ([4.4, 6.3] ^34^)
	LA_Pmax_ [mmHg]	[6, 20]^24^	12.8 ± 3.2 (n = 25)	–	–
	LA_Pmean_ [mmHg]	[4, 12]^24^	10.2 ± 2.8 (n = 27)	–	–
	LV_Pmax_ [mmHg]	[90, 140]^24^	124.4 ± 13.3 (n = 29)	–	–
	RA_I-Vmax_ [mL/m^2^]	[10, 36]^18^	31.8 ± 8.0 (n = 28)	–	[15, 29]^11^/[14, 25]^33^
	RV_I-EDV_ [mL/m^2^]	[44, 80]^19^	75.4 ± 12.4 (n = 29)	–	–
	RV_EF_ [%]	[44, 71]^19^	53.6 ± 5.3 (n = 29)	–	–
	SVR [mmHg min/L]	[11.3, 17.5]^24^	15.9 ± 3.3 (n = 29)	–	[8.1, 13.0]^11^
	PVR [mmHg min/L]	[1.9, 3.1]^24^	3.0 ± 1.4 (n = 28)	–	[3.1, 4.7]^11^
Sample size too small	LA_Pmin_ [mmHg]	[− 2, 9]^24^	7.4 ± 2.2 (n = 22)	–	–
	LV_Pmin_ [mmHg]	[4, 12]^24^	6.2 ± 1.4 (n = 12)	–	–
	RV_I-ESV_ [mL/m^2^]	[19, 46]^19^	33.1 ± 8.4 (n = 14)	–	–
	RA_Pmax_ [mmHg]	[2, 14]^24^	11.7 ± 3.3 (n = 9)	–	–
	RA_Pmin_ [mmHg]	[− 2, 6]^24^	4.4 ± 2.9 (n = 10)	–	–
	RA_Pmean_ [mmHg]	[− 1, 8]^24^	7.0 ± 2.6 (n = 23)	–	–
	RV_Pmin_ [mmHg]	[0, 8]^24^	3.0 ± 2.6 (n = 5)	–	–
	Shunt Fraction [%]	[0, 5]^7^	3.7 ± 0.8 (n = 9)	–	–

The mean and the standard deviation of samples are provided together with the sizes in brackets. If there is statistical evidence of impairments of the cardiocirculatory system associated with COVID-19 the p-value is below $0.01$. If the mean of a sample lied in the healthy range, the hypothesis test was not performed. The sample sizes less than $25$ were too small to perform the hypothesis tests. For some of the physical quantities with a big sample size, we report the COVID-19 ranges taken from literature. Test II.

## Discussion

This study addressed the association between COVID-19-related pneumonia and the impairments of the cardiovascular system. This has been faced by analysing clinical measures and model outputs computed through a calibrated lumped-parameter cardiocirculatory mathematical model. To the best of our knowledge, the current study is the first that used clinical measures and calibrated models to infer the cardiovascular physical quantities significantly altered in association with COVID-19-related pneumonia.

We start by discussing the available clinical data measured at L. Sacco Hospital in Milan and related to cardiovascular physical quantities for COVID-19 pneumonia patients. We found that none of the measured physical quantities (i.e. PQ1) was altered in association with COVID-19-related pneumonia (Table 1). See also ref.^31^ for another analysis of the same dataset of clinical measures.

Regarding the analysis of MO2, we noticed from Fig. 3 that some of the related physical quantities (among PQ2) lied within healthy ranges (e.g. LV_Pmax_), whereas other physical quantities lied outside them (e.g. RV_Pmax_). For the remaining physical quantities, we could not infer from Fig. 3 if they were altered or not in association with COVID-19-related pneumonia (e.g. PWP_mean_ or RA_Pmean_). Therefore, to significantly assess the alterations associated with COVID-19, we resorted to hypothesis tests.

We found that the pulmonary resistances (PVR), did not significantly increase in association with COVID-19-related pneumonia (Table 2). Nonetheless, we highlighted a slightly large value of PAP_max_ (Table 1) that was accompanied by a significant increase not only in PAP_min_, PAP_mean_ and RV_Pmax_, but also in PWP_min_ and PWP_mean_ (Table 2). These results seem to be in line with previous evidence reported in COVID-19-related pneumonia patients studied with cardiac catheterization^11^. In this study, patients did not show an increase in PVR but the mild increase in pulmonary arterial pressure was associated with an increase in wedge pressure. The authors hypothesized that a hyperdynamic state not accompanied by an increased in hypoxic-driven vasoconstriction could determine (especially in their population of old and often hypertensive patients) an increase in wedge pressure related to an increase of LV filling pressure. In our population a substantial percentage of patients were old ($34\%$ were older than $64$ years), with arterial hypertension and/or diabetes, conditions that could be in line with this interpretation, taking into account that the mean value of cardiac output computed by the model was rather large ($5.9\pm 1.0$ L/min, Table 2).

There was no statistical evidence that the maximal and mean left atrial pressures increased (Table 2). This could be due to limitations of the lumped-parameter model in representing the atria. Unfortunately, the sample size of LV_Pmin_ was too small to infer any interpretation.

In what follows, we refer to clinical literature of patients affected by COVID-19 for a comparison with the outcomes of our mathematical model (the model outputs MO2)^11,32–34^ (see Table 2). If the mean of our samples lied in the intervals identified in clinical literature, we considered them in accordance one another. We noticed from Table 2 that the sample mean of some of the physical quantities (RV_Pmax_, PAP_min_, PAP_mean_, PWP_mean_, LV_SV_ and CO) agreed with the COVID-19 literature, whereas the means of RA_I-Vmax_ and SVR were slightly larger and PVR slightly lower than the values of literature, although still lying inside the healthy range.

We emphasise that the statistical analysis of raw clinical data did not allow us to infer alterations in the cardiovascular system in association with COVID-19 infection (Table 1). Instead, thanks to the computational model we proposed, suitably calibrated by using the clinical data, we were able to identify some specific physical quantities related to pulmonary circulation (i.e. RV_Pmax_, PAP_min_, PAP_mean_, PWP_min_ and PWP_mean_) which were significantly altered in association with COVID-19, in the sense that there was a statistically relevant discrepancy with respect to the healthy ranges. This showed the importance of combining clinical data and computational models as an effective strategy to give meaningful insights about the impairments of the cardiocirculatory system associated with COVID-19 on cardiovascular physical quantities, which was not possible with raw clinical data and non-calibrated computational tools. Therefore, the proposed model-based approach has the advantage of increasing the interpretability of clinical data compared to signal processing methods, extrapolating information about physical quantities (PQ2) not referring to raw clinical data.

We now discuss the limitations of this study. First, notice that we did not have at disposal a control group to perform hypothesis tests in tests I–II, so we took a conservative approach comparing the mean of our samples with the lower and upper bounds of healthy ranges found in literature to infer the impairments of the cardiovascular system in association with the infection of COVID-19. The sample means of physical quantities significantly outside the corresponding healthy range highlight a clear impairment of a compartment of the cardiocirculatory system in association with COVID-19 infection. Nevertheless, we do not exclude that small changes in some physical quantity could indicate an impairment in the cardiocirculatory system as well.

Second, although being able to capture the considerable haemodynamic features, the lumped-parameter model is rather simple in comparison to other models for the study of the cardiac function (see e.g. refs.^35–38^). Improvements of the computational model will allow also to use other clinical measurements not used in this work (such as partial pressures of oxygen and carbon dioxide). In particular, we neglected cardio-respiratory interactions. Due to alterations of quantities of interest in the pulmonary circulation found by means of our analysis, the integration of the lumped-parameter cardiocirculatory model with a respiratory^38^ and a gas exchange model^35^ should be included in future works to provide further clinical insights concerning the hypoxemia typically caused by COVID-19-related pneumonia.

Third, to quantify the uncertainty in the estimation of the model outputs, we adopted a rather simple approach in terms of independence in the selection of the parameter configurations used to computationally generate the model outcomes. More sophisticated strategies that account for a selective choice of the new parameter configuration starting from the previous ones (e.g. Markov chain Monte Carlo methods^27^), which nevertheless entail a larger computational cost, could be considered in further developments of this work.

Possible improvements of the present work are also related to the clinical measurement acquisition. Other clinical measurements, when available, could be added to the framework of the present work to improve the outcomes. It may be of particular interest having a measure of the shunt fraction, that gives information on the pulmonary capillaries, to avoid the a priori assumption between micro-thrombosis and blunted hypoxic pulmonary vasoconstriction. As a limitation, in this work we neglected the contribution of micro-thrombosis^10^ and we focused only on the study of blunted hypoxic pulmonary vasoconstriction in the increase of non-oxygenated blood^11^.

Finally, we notice that the approach presented in this work can be extended to cardiovascular diseases different from COVID-19-related pneumonia, such as hypertension, patent foramen ovale or partial anomalous pulmonary venous return. To represent a different condition from COVID-19-related pneumonia, a modification of the compartments of the lumped-parameter model might be required. For example, in the case of patent foramen ovale, a circuit branch connecting the two atria should be added to the model^39^.

### Supplementary Information

Supplementary Information.

Supplementary Tables.

## Data Availability

The datasets generated and/or analysed are available from the corresponding author upon reasonable request.
