# Supplementary material for: A mathematical model to assess the effects of COVID-19 on the cardiocirculatory system
Source: Sci Rep. 2024 Apr 9;14:8304. doi: 10.1038/s41598-024-58849-3 (PMC11004160; doi:10.1038/s41598-024-58849-3)
Supplement: Supplementary file 1 — Supplementary Information. [file 41598_2024_58849_MOESM1_ESM.pdf]

A mathematical model to assess the effects of COVID-19 on the cardiocirculatory system

Andrea Tonini, Christian Vergara, Francesco Regazzoni, Luca Dede', Roberto Scrofani, Chiara Cogliati & Alfio Quarteroni

Supplementary Equations S1

The whole dynamical system for the lumped-parameter cardiovascular model is:

$$
\begin{cases}
\dot{V}_{\mathrm{LA}}(t) = Q^{\mathrm{PUL}}_{\mathrm{VEN}}(t) - Q_{\mathrm{MV}}(t) \\
\dot{V}_{\mathrm{LV}}(t) = Q_{\mathrm{MV}}(t) - Q_{\mathrm{AV}}(t) \\
C^{\mathrm{SYS}}_{\mathrm{AR}}\dot{p}^{\mathrm{SYS}}_{\mathrm{AR}}(t) = Q_{\mathrm{AV}}(t) - Q^{\mathrm{SYS}}_{\mathrm{AR}}(t) \\
L^{\mathrm{SYS}}_{\mathrm{AR}}\dot{Q}^{\mathrm{SYS}}_{\mathrm{AR}}(t) = -R^{\mathrm{SYS}}_{\mathrm{AR}}Q^{\mathrm{SYS}}_{\mathrm{AR}}(t) + p^{\mathrm{SYS}}_{\mathrm{AR}}(t) - p^{\mathrm{SYS}}_{\mathrm{C}}(t) \\
C^{\mathrm{SYS}}_{\mathrm{C}}\dot{p}^{\mathrm{SYS}}_{\mathrm{C}}(t) = Q^{\mathrm{SYS}}_{\mathrm{AR}}(t) - Q^{\mathrm{SYS}}_{\mathrm{C}}(t) \\
C^{\mathrm{SYS}}_{\mathrm{VEN}}\dot{p}^{\mathrm{SYS}}_{\mathrm{VEN}}(t) = Q^{\mathrm{SYS}}_{\mathrm{C}}(t) - Q^{\mathrm{SYS}}_{\mathrm{VEN}}(t) \\
\dot{Q}^{\mathrm{SYS}}_{\mathrm{VEN}}(t) = -R^{\mathrm{SYS}}_{\mathrm{VEN}}Q^{\mathrm{SYS}}_{\mathrm{VEN}}(t) + p^{\mathrm{SYS}}_{\mathrm{VEN}}(t) - p_{\mathrm{RA}}(t) \\
\dot{V}_{\mathrm{RA}}(t) = Q^{\mathrm{SYS}}_{\mathrm{VEN}}(t) - Q_{\mathrm{TV}}(t) \\
\dot{V}_{\mathrm{RV}}(t) = Q_{\mathrm{TV}}(t) - Q_{\mathrm{PV}}(t) \\
C^{\mathrm{PUL}}_{\mathrm{AR}}\dot{p}^{\mathrm{PUL}}_{\mathrm{AR}}(t) = Q_{\mathrm{PV}}(t) - Q^{\mathrm{PUL}}_{\mathrm{AR}}(t) \\
L^{\mathrm{PUL}}_{\mathrm{AR}}\dot{Q}^{\mathrm{PUL}}_{\mathrm{AR}}(t) = -R^{\mathrm{PUL}}_{\mathrm{AR}}Q^{\mathrm{PUL}}_{\mathrm{AR}}(t) + p^{\mathrm{PUL}}_{\mathrm{AR}}(t) - p^{\mathrm{PUL}}_{\mathrm{C}}(t) \\
(C_{\mathrm{SH}} + C^{\mathrm{PUL}}_{\mathrm{C}})\dot{p}^{\mathrm{PUL}}_{\mathrm{C}}(t) = Q^{\mathrm{PUL}}_{\mathrm{AR}}(t) - Q_{\mathrm{SH}}(t) - Q^{\mathrm{PUL}}_{\mathrm{C}}(t) \\
C^{\mathrm{PUL}}_{\mathrm{VEN}}\dot{p}^{\mathrm{PUL}}_{\mathrm{VEN}}(t) = Q_{\mathrm{SH}}(t) + Q^{\mathrm{PUL}}_{\mathrm{C}}(t) - Q^{\mathrm{PUL}}_{\mathrm{VEN}}(t) \\
L^{\mathrm{PUL}}_{\mathrm{VEN}}\dot{Q}^{\mathrm{PUL}}_{\mathrm{VEN}}(t) = -R^{\mathrm{PUL}}_{\mathrm{VEN}}Q^{\mathrm{PUL}}_{\mathrm{VEN}}(t) + p^{\mathrm{PUL}}_{\mathrm{VEN}}(t) - p_{\mathrm{LA}}(t)
\end{cases}
$$

coupled with suitable initial conditions.

The blood flows across cardiac valves are:

$$
Q_{\mathrm{TV}}(t) = Q_{\mathrm{valve}}(p_{\mathrm{RA}}(t) - p_{\mathrm{RV}}(t)), \quad Q_{\mathrm{MV}}(t) = Q_{\mathrm{valve}}(p_{\mathrm{LA}}(t) - p_{\mathrm{LV}}(t)),
$$
$$
Q_{\mathrm{PV}}(t) = Q_{\mathrm{valve}}(p_{\mathrm{RV}}(t) - p^{\mathrm{PUL}}_{\mathrm{AR}}(t)), \quad Q_{\mathrm{AV}}(t) = Q_{\mathrm{valve}}(p_{\mathrm{LV}}(t) - p^{\mathrm{SYS}}_{\mathrm{AR}}(t)),
$$

where

$$
Q_{\mathrm{valve}}(\Delta p) = \frac{\Delta p}{R_{\mathrm{valve}}(\Delta p)},
$$
$$
R_{\mathrm{valve}}(\Delta p) = R_{\mathrm{min}}^{\frac{1}{2}-\frac{1}{\pi}\arctan(-100\pi\Delta \mathrm{p})} R_{\mathrm{max}}^{\frac{1}{2}+\frac{1}{\pi}\arctan(-100\pi\Delta \mathrm{p})}.
$$

and the pressure inside each cardiac chamber $c \in \{LA, LV, RA, RV\}$ is:

$$
\begin{aligned}
p_{\mathrm{c}}(t) &= E_{\mathrm{c}}(t)(V_{\mathrm{c}}(t) - V0_{\mathrm{c}}), \\
E_{\mathrm{c}}(t) &= EB_{\mathrm{c}} + EA_{\mathrm{c}} e_{\mathrm{c}}(t),
\end{aligned}
$$

$$
e_{\mathrm{c}}(t) = \begin{cases} \frac{1}{2}\left[1 - \cos\left(\frac{\pi}{TC_{\mathrm{c}}} \mathrm{mod}\,(t - tC_{\mathrm{c}}, T_{\mathrm{HB}})\right)\right] & \text{if } 0 \leq \mathrm{mod}\,(t - tC_{\mathrm{c}}, T_{\mathrm{HB}}) < TC_{\mathrm{c}}, \\ \frac{1}{2}\left[1 + \cos\left(\frac{\pi}{TR_{\mathrm{c}}} \mathrm{mod}\,(t - tR_{\mathrm{c}}, T_{\mathrm{HB}})\right)\right] & \text{if } 0 \leq \mathrm{mod}\,(t - tR_{\mathrm{c}}, T_{\mathrm{HB}}) < TR_{\mathrm{c}}, \\ 0 & \text{otherwise.} \end{cases}
$$

The blood flows in capillaries are:

$$
\begin{aligned}
R_{\mathrm{C}}^{\mathrm{SYS}} Q_{\mathrm{C}}^{\mathrm{SYS}}(t) &= p_{\mathrm{C}}^{\mathrm{SYS}}(t) - p_{\mathrm{VEN}}^{\mathrm{SYS}}(t), \\
R_{\mathrm{C}}^{\mathrm{PUL}} Q_{\mathrm{C}}^{\mathrm{PUL}}(t) &= p_{\mathrm{C}}^{\mathrm{PUL}}(t) - p_{\mathrm{VEN}}^{\mathrm{PUL}}(t), \\
R_{\mathrm{SH}} Q_{\mathrm{SH}}(t) &= p_{\mathrm{C}}^{\mathrm{PUL}}(t) - p_{\mathrm{VEN}}^{\mathrm{PUL}}(t),
\end{aligned}
$$
